# The subjective experience of young women with non-metastatic breast cancer: the Young Women with Breast Cancer Inventory

**DOI:** 10.1186/s12955-015-0273-x

**Published:** 2015-06-03

**Authors:** V. Christophe, C. Duprez, A. Congard, P. Antoine, A. Lesur, E. Fournier, L. Vanlemmens

**Affiliations:** Université de Lille – SCALab UMR CNRS 9193, Rue du Barreau, BP 60149, F-59653 Villeneuve d’Ascq cedex, France; SIRIC ONCOLille - Maison Régionale de la Recherche Clinique - 6, rue du Professeur Laguesse, 59037 Lille cedex, France; Aix-Marseille Université, Centre de Recherche PsyCLÉ (EA 3273), 29, avenue Robert Schuman, F-13621 Aix en Provence cedex 1, France; Institut de Cancérologie de Lorraine- Centre Alexis Vautrin, 6 Avenue de Bourgogne, 54519 Vandoeuvre-lès-Nancy, France; Centre Oscar Lambret - Département de Sénologie, BP 307, F-59020 Lille cedex, France

**Keywords:** Breast cancer, Quality of life, Subjective experience, Young women, Health psychology

## Abstract

**Background:**

The subjective experience of young women with breast cancer has some particular features linked to the impact of the disease and its treatment on their age-related issues (e.g. desire for a child, couple relationship, career management). Despite these specific concerns, no questionnaire currently targets the young breast cancer patient’s quality of life, subjective experience or common problems when facing cancer. This study presents the psychometric validation of an inventory that aimed to measure the impact of breast cancer on the quality of life of young women (<45 years of age) with non-metastatic disease.

**Methods:**

546 women aged <45 years when diagnosed with a non-metastatic breast cancer were recruited in 27 French cancer research and treatment centers. They answered a self-reported questionnaire created from verbatim collected by non-directive interviews carried out with 69 patients in a first qualitative study. Exploratory and confirmatory analyses were conducted in order to obtain the final structure of the scale. Internal consistency, test-retest reliability and concurrent validity with quality of life questionnaires currently used (QLQ-C30 and the QLQ-BR23 module) were then assessed.

**Results:**

The YW-BCI36 contains 36 items and highlights 8 factors: 1) *feeling of couple cohesion,* 2) *negative affectivity and apprehension about the future*, 3) *management of child(ren) and of everyday life,* 4) *sharing with close relatives*, 5) *body image and sexuality*, 6) *financial difficulties*, 7) *deterioration of relationships with close relatives*, and 8) *career management*. Psychometric analyses indicated good internal consistency (Cronbach’s alpha values ranging from 0.76 to 0.91) and temporal reliability (Bravais-Pearson correlations ranging from 0.66 to 0.85). As expected, there were quite strong correlations between the YW-BCI36 and the QLQ-C30 and QLQ-BR23 scores (*r* ranging from 0.20 to −0.66), indicating adequate concurrent validity.

**Conclusions:**

The YW-BCI36 was confirmed as a valid scale for evaluating the subjective experience of breast cancer in young women. This instrument could help to identify the problems of these women more precisely, in order to respond to them better by an optimal care management. This scale may improve the medical, psychological and social care of breast cancer patients.

## Background

The study of quality of life in the health area has considerably grown in recent decades. Defined by the WHO in 1994, quality of life refers to the “individuals perception of their position in life in the context of the culture and value systems in which they live and in relation to their goals, expectations, standards and concerns ” [1]. This is above all a measure of quality of life in relation with health in general, health being defined as a “state of complete physical, mental, and social well-being, and not merely the absence of disease” [1]. Well-being is in turn defined as having two dimensions, subjective and objective, and “comprises an individual’s experience of their life as well as in comparison of life circumstances with social norms and values” [2]. Most authors thus agree that quality of life is a concept that encompasses three key dimensions: physical, psychological and social e.g. [3]. With this conceptual anchor, it therefore seems essential to take into account the specificities, standards, environmental and societal values of the health and/or disease context in which individuals live, in order to more precisely target the specificities of their subjective and objective experience.

In oncology, novel therapeutics have greatly improved the survival rate of women with cancer, but they often entail significant side effects that worsen the patient’s physical and psychological quality of life, at every stage of their care pathway, from the diagnosis announcement to the follow-up or “survivorship” period [4–6]. Health-related quality of life (HRQOL) is defined as the physical, psychological and social responses of the patient when facing the disease and its treatment [7, 8] as well as the patient’s subjective perception of the impact of the cancer and its treatment on different areas of his/her life: physical, functional, emotional and social [9].

Evaluating quality of life appears crucial in everyday clinical practice for detecting physical and emotional problems provoked by the disease or its treatment. This contributes to improving patients’ care by guiding them, according to their identified problems and needs, toward appropriate support care, throughout their care pathway [7, 10–12]. Every woman suffering from breast cancer has to face many issues e.g. [13–16], but the consequences of the disease and its treatment are markedly different and have specific features in young women (under 45–50 years old at the time of the initial diagnosis), who represent almost 13% of diagnosed breast cancer cases [17, 18]. Some indicators were identified in the literature as most useful for using the age criterion of 45 or younger as it applies to breast cancer: the woman is of child bearing age, the woman has young child(ren), that is, child(ren) not yet at secondary school; or the woman is premenopausal [18, 19]. These women are confronted by issues that are specific to their age, like (i) the fact that they cannot be sure of seeing their child(ren) grow up or have problems in bringing them up, all the more so as their child(ren) is/are very young; (ii) the quality of their marital relationship and sexuality and (iii) their current or future professional career for reviews, see [4, 20–23]. These specific issues, in addition to the physical consequences of the disease and its treatment, could partly explain why young women show higher emotional distress levels, less quality of life, and more problems in setting up adjustment strategies to face the disease than older women do e.g. [24–28].

The assessment instruments that are most frequently used, such as the QLQ-C30 coupled with the module especially developed for breast cancer QLQ-BR23 [29], the FACT-G coupled with the module FACT-B for breast cancer [30], or the unmet needs assessment tools for reviews, see [31, 32], aim to evaluate the quality of life of women with breast cancer as a whole, whatever their age. To our knowledge, there is as yet no assessment tool to evaluate the specific subjective experience of young women living with breast cancer. Following the example of some authors [33, 34], it seems crucial to consider all the life areas that may be affected by the disease experience, in a given context governed by norms and specific values. For this, it is essential to have appropriate tools, created from the experiences of the patients themselves -in order to be as close as possible to their subjective experience-, presenting the required psychometric properties and easily usable by the medical caregivers in the care pathway. To create such a tool thus seems particularly relevant for helping clinicians to identify the issues specific to this population and, using the results provided by the tool, to guide their patients toward appropriate social support services, at every stage of their care pathway [12, 31, 35–37].

The present study aims to validate, in a large sample of patients, a questionnaire specifically measuring the subjective experience of the disease and its treatment in young women (under 45 years old when diagnosed) living with a non-metastatic breast cancer and the repercussions of the disease and its treatment they perceive in their daily life. This scale (“Young Women with Breast Cancer Inventory - YW-BCI”) differs by being created from non-directive interviews carried out with patients in a previous qualitative study [38, 39], and by taking into account the specific features of women facing breast cancer at a young age. To better understand the temporal evolution of the cancer impact from the diagnosis announce to the follow-up period, four independent groups of patients were formed: 1. During the chemotherapy with or without Trastuzumab; 2. Under Trastuzumab with or without hormone therapy; 3. During the hormone therapy alone; and 4. During the follow-up period (after the end of all treatments).

## Methods

### Study design and population

Our data are part of an observational prospective multicenter study conducted from January 2010 to June 2012 in 27 French cancer treatment and research centers and involving 772 young women with diagnoses of non-metastatic breast cancer. Out of the 772 patients to whom the study was proposed, 546 patients agreed to participate and returned their filled questionnaire (168 undergoing chemotherapy with or without Trastuzumab, 58 undergoing Trastuzumab with or without hormone therapy, 176 patients undergoing hormone therapy only, and 144 under follow-up, after the end of treatment). Patients were included by their oncologist if they were aged less than 45 years at their diagnosis for a non-metastatic breast cancer, had received or were receiving chemotherapy, and had signed an informed consent form. Patients with poor knowledge of the French language were not included in the study.

The objectives and procedure of the study were explained to the patients during an outpatient appointment. After they had agreed orally and signed the consent form, patients received a questionnaire and a socio-demographic data form. They were asked to complete these documents in a quiet location and to return them in a pre-stamped envelope to the research centers. Conditions of anonymity and confidentiality were guaranteed. At the same time, patients’ medical data were collected by each investigator in the participating centers. All women were asked to complete the YW-BCI. To test concurrent validity, 111 women also answered a standard quality of life questionnaire (QLQ-C30 and QLQ-BR23, [29]). For the reliability analysis, the YW-BCI was administered two weeks after the baseline measurement in 116 patients. The distribution in one of these 3 groups (YW-BCI only, YW-BCI + QLQ, YW-BCI two times) was randomly made at the time the patients entered in the study.

This study was performed in accordance with the Declaration of Helsinki and was approved by the Ethics Committee for the Protection of Persons, the Consultative Committee for Data Processing in Research in the Field of Health, and the National Commission for Data Protection.

### Measures

#### YW-BCI

The YW-BCI scale consists of 80 items resulting from a previous qualitative study carried out with 69 young patients (<45 years old at the time of diagnosis) living with a non-metastatic breast cancer [38, 39]. Items were produced from a thematic content analysis with inter-judges agreement and were semantically validated by 15 couples who participated in these interviews [38, 39].

From this first qualitative step, 80 items were selected and organized into 8 distinct dimensions: 1) *psychological and affective experience* concerning the present and the future (14 items, e.g. emotions felt, perception of the future), 2) *physical experience* (8 items, e.g. physical side effects of treatments, fatigue, body image perception), 3) *management of daily life* (5 items, e.g. problems in managing housework and daily life, limitations felt in doing housework, changes in life habits), 4) *questions about the child(ren)* (8 items, e.g. problems in managing the education of the child(ren), fear for the chil(dren), availability, communication), 5) *professional life* (4 items, e.g. sick leave, personal investment in job, taking days off, effectiveness at work, career, professional difficulties), 6) *financial difficulties* (4 items, e.g. income decrease, problems in getting a loan, additional costs), 7) *familial and social relationships* (16 items, e.g. communication, cohesion, social support, perception of the impact of the disease on relatives and on social activities), and 8) *couple relationship* (21 items, e.g. communication, cohesion and social support within the couple, impact of the disease on the partner, sexuality).

In order to create and validate an inventory that shows good psychometric properties, the patients in the present study were instructed to indicate to what extent each of the 80 assertions corresponded to their current state (“at this moment, currently”), using a 5-point Likert scale, from 1: *“strongly disagree”* to 5: *“strongly agree”.*

#### EORTC QLQ-C30

In order to test the concurrent validity of the YW-BCI, the patients were invited to complete the French version of the EORTC QLQ-C30 (version 3, [29]).This self-reported questionnaire aims to evaluate the health-related quality of life of cancer patients, whatever the cancer site, using 2 dimensions: the overall functioning and the symptoms felt. In line with the EORTC guidelines, each score was linearly transformed, so that it varied from 0 to 100. The higher the functioning scale score is, the better the functioning level of the patient is. On the contrary, an elevated score on the symptoms scale indicates a greater presence of symptoms.

#### EORTC QLQ-BR23

The patients were also invited to complete the EORTC QLQ-BR23, a module developed to assess the particular features of the quality of life of breast cancer patients. This module contains 23 items that enable the repercussions of the disease and the side effects of its treatment to be measured. For each dimension, the scores vary from 0 to 100. As for the EORTC QLQ-C30, a high score on the functioning scale indicates a high level of functioning, whereas for the symptoms scale, a high score indicates a high level of symptoms.

### Statistical analysis

The construct validity of the scale was assessed by exploratory and confirmatory analyses. The exploratory principal component analysis (PCA) were conducted on a sample of 304 patients, randomly chosen from the 546 patients who completed the instrument. The confirmatory analyses (CFA) were carried out on a random sample of 250 patients from this initial sample.

Descriptive analyses (mean, standard deviation, skewness and kurtosis) were conducted first, in order to eliminate the poorly discriminative items (*n* = 14). Exploratory principal component analyses using the principal axis factoring method of extraction and Promax rotation were then conducted with the Statistical Package for Social Sciences Version 18 to identify the factor structure of the scale. At this step, items showing cross-loadings or loadings less than 0.30 were eliminated (*n* = 32). Items were thus selected with factor loadings of at least 0.30. Series of confirmatory factor analyses based on a structural equation modeling method were then conducted on the retained items, with LISREL 8.8 software [40].

Internal consistency was examined for each subscale of the YW-BCI by using Cronbach’s alpha coefficients. Test-retest reliability was investigated using the Bravais-Pearson’s correlation coefficient for the sample of patients (*n* = 116) who completed the YW-BCI questionnaire twice.

The convergent validity of our scale was assessed by calculating the Bravais-Pearson’s correlation coefficients between the patients’ scores in the YW-BCI and their scores in the QLQ-C30 and QLQ-BR23. Given that they were subjective quality of life constructs, quite strong correlations were expected between the subjective dimensions of quality of life measured by the QLQ-C30 and QLQ-BR23 (emotional functioning, social functioning, etc.) and the subjective dimensions that constitute the YW-BCI (Pearson’s *r* > 0.50). Weaker correlations were expected between these subjective dimensions and the quality of life dimensions involving the physical consequences of the disease and its treatment (Pearson’s *r* < 0.40).

## Results

### Sample characteristics

The sample included 546 young women, aged 23–62 (*M* = 40.64, *SD* = 6.21). On average, patients who participated in the study had suffered from breast cancer for 2.47 years (*SD* = 2.79, time since diagnosis ranging from 21 days (Min) to 19.05 years (Max)). The majority of patients had a job (88.8%), more than half of them were on sick leave or were working part-time for medical reasons when the study was carried out. Almost half of them had post-secondary education or higher education qualifications (48.79%). The majority of the patients (97.80%) were living in a couple at the time of diagnosis (*M*_*years*_ = 13.71, *SD* = 8.37 years), and had a child or children (88.6%) (Table 1).

**Table 1:** Patients characteristics (*N* = 546)

Demographics	
Age (Range, Mean, SD)	23-62 (40.64, 6.21)
Employment	
Farmers	5
Craftspersons, shopkeepers, company managers	28
Executives and intellectual professions	69
Intermediate professions	106
Employees	253
Workers	17
Retired	2
Unemployed	58
Missing	8
Education	
No certificate	34
Secondary education diploma – below baccalaureate	129
Baccalaureate or equivalent diploma	114
First undergraduate cycle degree or equivalent diploma	119
Second university cycle degree, doctorate or equivalent diploma	145
Missing	5
Presence of child(ren)	484
Length of the relationship (in years): Mean *(*SD)	13.71 (8.37)
Disease and treatments characteristics	
Event type	
First breast cancer	517
Local recurrence	14
Contralateral cancer	13
Missing	2
Time since diagnosis (in years): Mean (SD)	2.47 (2.79)
Treatment for the current event^a^	
Surgery	
Yes	499
No but planned	42
No	5
Partial mastectomy	311
Total mastectomy	213
Immediate reconstruction	21
Delayed reconstruction	132
Unwanted reconstruction	17
Unknown	11
sentinel lymph node biopsy	160
axillary lymph node dissection	359
Radiotherapy	
Yes	343
No but planned	142
No	50
Missing	11
Trastuzumab	
Yes	134
No but planned	11
No	397
Missing	4
Hormone therapy	
Yes	251
No but planned	131
No	163
Missing	1
*Tamoxifen*	229
*Gonadotropin-Releasing Hormone (GnRH)*	29
*Aromatase inhibitors*	29
Psychotherapy	
Yes	74
No	466
Missing	6
Antidepressants or anxiolytic drugs	
Yes	148
No	394
Missing	4
Sick leave	
Yes	236
No	282
Non applicable	23
Working part-time for medical reasons	
Yes	28
No	481
Non applicable	29
Missing	8

^a^All the patients were or had been undergoing chemotherapy at the time of the study

At the medical level, for most patients it was their first cancer (94.7%), 91.4% had had a mastectomy: 62.3% had had a partial mastectomy with axillary node dissection for 71.9% of them while 42.7% of the patients had had a total mastectomy. For 61.97% of these patients who had had a total mastectomy, a delayed reconstruction was planned. Lastly, 74 patients had participated in psychotherapy when the study was conducted, and 148 patients had received antidepressants or anxiolytic drugs.

### Factor structure of the YW-BCI questionnaire

#### Exploratory analyses

A structure of 8 factors emerged from the exploratory principal component analysis which was preceded by the analysis of the graph of eigenvalues -for determining the optimal number of dimensions-, and the theoretical relevance of the constructs that appeared. These factors explained 57.24% of the total variance and were as follows: 1) *feeling of couple cohesion* (19.78% of the total variance), 2) *negative affectivity and apprehension about the future* (12.52%), 3) *management of child(ren) and of everyday life* (7.81%), 4) *sharing with close relatives* (4.83%), 5) *body image and sexuality* (4.41%), 6) *financial difficulties* (3.17%), 7) *deterioration of relationships with close relatives* (2.46%), and 8) *career management* (2.25%) (Table 2).

**Table 2:** Factor structure of the questionnaire (loadings)

Items	Feeling of couple cohesion	Negative affectivity and apprehension about the future	Management of child(ren) and of everyday life	Sharing with close relatives	Body image and sexuality	Financial difficulties	Deterioration of relationships with close relatives	Career management
I feel close to my partner. (Je me sens proche de mon (ma) partenaire)	0.920							
My couple is strong. (Mon couple est uni)	0.895							
I feel supported by my partner. (Je me sens soutenue par mon (ma) partenaire)	0.850						−0.266	
My partner helps me a lot. (Mon (ma) partenaire m’aide beaucoup)	0.785						−0.296	
I can confide in my partner. (Je peux me confier à mon (ma) partenaire)	0.718							
I feel worried. (Je me sens inquiète)		0.826				0.244		
I have anxieties. (J’ai des angoisses)		0.794						
I’m concerned about the future. (L’avenir me préoccupe)		0.639						
I am calm. (Je suis sereine) (R)		−0.637	−0.369		−0.339		−0.388	
I think about my disease every day. (Je pense tous les jours à la maladie)		0.543			0.312			
I am afraid for my child(ren). (J’ai peur pour mon (mes) enfant(s))		0.533						
I have trouble taking care of the home. (J’ai des difficultés pour m’occuper du logement)		0.315	0.810		0.339	0.262		0.327
I have problems managing daily life with my child(ren). (J’ai des difficultés dans la gestion du quotidien avec mon (mes) enfant(s))		0.347	0.753			0.345		0.383
I feel that I’m neglecting managing daily life. (J’ai l’impression de négliger la gestion du quotidien)		0.301	0.663		0.311	0.353		0.387
I delegate most daily tasks. (Je délègue la majorité des tâches du quotidien)			0.644			0.313		0.351
I have problems managing the education of my child(ren). (J’ai des difficultés à gérer l’éducation de mon (mes) enfant(s))			0.606			0.204		0.289
I can confide in some people. (Je peux me confier à certaines personnes)				0.791			−0.327	
Some people around me help me a lot. (Certaines personnes de mon entourage m’aident beaucoup)				0.788			−0.311	
I talk about my disease with those around me. (Je discute de la maladie avec mon entourage)				0.772				
I talk about how I feel with some of my close relatives. (Je parle de ce que je ressens à certains de mes proches)				0.677				
I feel less sexual desire. (Je ressens moins de désir sexuel)			0.329		0.823			
I have some sexual problems because of my disease. (Je rencontre des difficultés sexuelles à cause de la maladie)					0.809			
I have trouble letting my partner touch me physically. (J’ai des difficultés à accepter que mon (ma) partenaire me touche physiquement)		0.319			0.682			
I feel that I’m neglecting my partner. (J’ai l’impression de délaisser mon (ma) partenaire).	−0.305		0.358		0.676			
I have problems dealing with the costs incurred by my disease. (J’ai des difficultés pour faire face aux frais engendrés par la maladie)		0.311	0.256			0.858	0.376	
I have financial difficulties. (J’ai des difficultés financières)			0.303			0.753		
My disease causes additional costs. (La maladie occasionne des frais supplémentaires)			0.399			0.665		0.356
I have to reduce my lifestyle. (Je dois diminuer mon train de vie)		0.301	0.205			0.510		0.261
I can count on those around me. (Je peux compter sur ceux qui m’entourent) (R)	0.302			0.331			−0.686	
I feel neglected by some of my close relatives. (Je me sens délaissée par certains de mes proches).	−0.313						0.665	
My disease creates tensions with the people around me. (La maladie crée des tensions avec les personnes qui m’entourent)		0.308	0.306		0.327	0.344	0.597	
I have problems communicating with some of my close relatives. (J’ai des problèmes de communication avec certains de mes proches)	−0.372	0.376		−0.339			0.589	
My disease has a negative impact on my close relatives. (La maladie a un impact négatif sur mes proches)							0.522	
I feel that I’m effective at work. (Je me sens efficace dans mon activité professionnelle) (R)			−0.392			−0.327		−0.820
I have problems doing my job. (J’ai des difficultés à exercer mon activité professionnelle).			0.377			0.392		0.815
I put a lot into my work. (Je m’investis dans mon travail) (R)			−0.381					−0.790
% of variance before rotation	7.12	4.508	2.81	1.74	1.59	1.14	0.884	0.812
% of variance after rotation	19.78	12.52	7.81	4.83	4.41	3.17	2.46	2.25
% added up after rotation	19.78	32.31	40.11	44.94	49.356	52.53	54.98	57.24

Loadings and proportions of variance reported are from a Principal Component Analysis (PCA) and Promax method for rotation. Loadings above 0.30 are shown in boldface. For the sake of readability, loadings below 0.20 are not shown

The final inventory, “YW-BCI36”, thus contained 36 items. The dimensions relating to *psychological and affective experience* concerning the present and the future, *management of daily life*, now completed by items linked with *questions about the child(ren), professional life* and *financial difficulties*, which were present in the initial version of the questionnaire, still remained. The dimension in line with *social and familial relationships* was split into two parts, depending on the positive or negative valence of the interpersonal relationships: a first dimension that was linked with social sharing, and a second dimension that was in line with the deterioration of relationships with close relatives. Likewise, two dimensions emerged related to the couple: *couple cohesion,* with items linked with support and closeness, and *body image and sexuality* with items that were more linked with couple intimacy.

These dimensions appeared to be intercorrelated (Table 3). Like for the majority of health-related quality of life questionnaires, the correlations were quite strong between the subscales and particularly for the *negative affectivity and apprehension about the future* and *management of the child(ren) and of everyday life* dimensions.

**Table 3:** Correlations between the 8 dimensions of the factor structure of the questionnaire

	1	2	3	4	5	6	7	8
F1. Feeling of couple cohesion	-							
F2. Negative affectivity and apprehension about the future	−0.19	-						
F3. Body image and sexuality	−0.33**	0.44**	-					
F4. Career management	0.08	−0.29**	−0.30**	-				
F5. Deterioration of relationships with close relatives	−0.36**	0.38**	0.35**	−0.22*	-			
F6. Management of child(ren) and of everyday life	−0.14	0.34**	0.42**	−0.49**	0.39**	-		
F7. Financial difficulties	−0.16	0.33**	0.30**	−0.39**	0.37**	0.48**	-	
F8. Sharing with close relatives	0.23*	−0.17	−0.17	−0.11	−0.37**	−0.05	−0.06	-

Bravais-Pearson’s *r* (*N* = 436). Correlations in boldface *p* < 0.001

#### Confirmatory analyses

The structure thus revealed was supported by the confirmatory factor analyses conducted on data from a random sample of 120 patients. The results generally suggested a good adjustment of the model ((χ^2^(585) = 987.793; p < 0.001; χ^2^/ddl = 1.68; RMSEA = 0.047; NFI = 0.91; SRMR = 0.070 ; NNFI = 0.96)

### Internal consistency and test-retest reliability

Cronbach’s alphas (Table 4) were acceptable overall in light of the number of items composing the subscales of the questionnaire (all were higher than 0.75, ranging from 0.76 to 0.91). The Bravais-Pearson’s correlation coefficients between the scores of the 116 patients who completed the questionnaire twice (mean time between the two completions: 16.57 days, *SD* = 5.03) showed that the test-retest reliability of the questionnaire was acceptable, with correlation coefficients ranging from 0.66 (for the dimension *sharing with close relatives*) to 0.85 (*feeling of couple cohesion*, Table 4).

**Table 4:** Internal consistency (Cronbach’s alpha) and test-retest reliability (Bravais-Pearson coefficient) of the questionnaire

	Number of items	Alpha	R
Feeling of couple cohesion	5	0.91	0.85
Negative affectivity and apprehension about the future	6	0.84	0.80
Body image and sexuality	4	0.82	0.85
Career management	3	0.85	0.77
Deterioration of relationships with close relatives	5	0.76	0.80
Management of child(ren) and of everyday life	5	0.81	0.79
Financial difficulties	4	0.80	0.82
Sharing with close relatives	4	0.83	0.66

For the test-retest reliability, all the correlations are significant at *p* < 0.01, *n* = 116 patients for whom the period between T1 and T2 was less than or equal to 1 month

### Concurrent validity

The *financial difficulties* dimension of the YW-BCI36 was strongly and positively correlated with the *financial difficulties* item of the QLQ-C30 (*r* = 0.73, *p* < 0.001) and the *negative affectivity and apprehension about the future dimension* of our scale was correlated with the *emotional functioning* dimension of the QLQ-C30 (*r* = −0.59, *p* < 0.001), suggesting that both questionnaires examined dimensions close to each other. The *management of child(ren) and of everyday life* dimension was further correlated with the *social functioning* dimension of the QLQ-C30 (*r* = −0.62, *p* < 0.001). Concerning the concurrent validity of the YW-BCI36 with the QLQ-BR23 module, the *body image and sexuality* dimension of the YW-BCI36 was correlated with the *sexual functioning*, *sexual enjoyment*, and *body image* subscales of the module, at −0.74, −0.65, and −0.41, respectively (*p* < 0.001), which indicated a fair convergence between these factors. In the same way, the *negative affectivity and apprehension about the future* subscale of the YW-BCI36 was negatively and moderately correlated with the *future perspective* dimension of the QLQ-BR 23 (*r* = −0.66, *p* < 0.001), see Table 5.

**Table 5:** Convergent validity of the YW-BCI36 with the functioning and symptoms scales of the QLQ-C30 and QLQ-BR23

Dimensions of the YW-BCI36	Feeling of couple cohesion	Negative affectivity and apprehension about the future	Body image and sexuality	Career management	Deterioration of relationships with close relatives	Management of child(ren) and of everyday life	Financial difficulties	Sharing with close relatives
EORTC QLQ-C30 functioning								
Overall health status	0.28*	−0.36**	−0.35**	0.61**	−0.35**	−0.63**	−0.43**	0.14
Physical functioning	0.16	−0.22*	−0.33**	0.52**	−0.32**	−0.60**	−0.45**	0.08
Role functioning	0.14	−0.31**	−0.15	0.51**	−0.24*	−0.58**	−0.34**	−0.02
Emotional functioning	0.21*	−0.59**	−0.41**	0.28*	−0.35**	−0.50**	−0.35**	0.21*
Cognitive functioning	0.35**	−0.27*	−0.20*	0.33**	−0.33**	−0.53**	−0.30**	0.05
Social functioning	0.20*	−0.30**	−0.29**	0.48**	−0.30**	−0.62**	−0.46**	−0.01
Financial difficulties	−0.28*	0.02	0.11	−0.42**	0.05	0.28**	0.73**	0.14
QLQ-BR23 functioning								
Body image	0.12	−0.41**	−0.41**	0.39**	−0.19	−0.31*	−0.31*	0.14
Sexual functioning	0.51**	−0.37**	−0.74**	0.29*	−0.17	−0.33	0.04	0.05
Sexual enjoyment	0.03	−0.33**	−0.65**	0.23*	−0.48**	−0.43**	−0.07	0.27
Future perspective	0.11	−0.66**	−0.32*	0.16	−0.21*	−0.25	−0.13	0.16
QLQ-C30 symptoms								
Fatigue	−016	0.31**	0.32**	−0.53**	0.20*	0.58**	0.52**	0.02
Nausea and vomiting	−0.22*	0.23*	0.18	−0.22*	0.13	0.35**	0.24*	0.04
Pain	−0.07	0.26*	0.18	−0.37**	0.19	0.51**	0.42**	0.02
Dyspnea	−0.01	0.09	0.02	−0.36**	0.19	0.29**	0.19	−0.19
Insomnia	−0.21*	0.42**	0.35**	−0.17	0.28*	0.46**	0.26**	−0.16
Appetite loss	−0.13	0.31**	0.33**	−0.26*	0.16	0.45**	0.33**	0.04
Constipation	−0.02	0.24*	0.25**	−0.24*	0.12	0.16	0.07	−0.10
Diarrhea	−0.10	0.18	0.10	−0.10	0.06	0.15	0.05	0.001
QLQ-BR23 symptoms								
Systemic therapy side effects	−0.08	0.37**	0.22	−0.49**	0.08	0.42**	0.35*	0.11
Breast symptoms	−0.49**	0.38**	0.21	−0.39**	0.37**	0.17	0.08	0.23
Arm symptoms	0.11	−0.09	−0.19	-	−0.22	−0.05	0.10	0.17
Upset by hair loss	−0.11	0.27*	0.39**	−0.18	−0.03	0.03	0.08	0.11

*p < 0.01; **p < 0.001

Concerning the concurrent validity with the symptoms scales, the results mainly evidenced positive and quite strong correlations between problems in the *management of the child(ren) and of everyday life* in the YW-BCI36 and almost all the symptoms, as well as negative correlations between some symptoms (fatigue, pain, dyspnea) assessed by the QLQ-C30 and difficulties in the *career management* dimension of the YW-BCI36 (Table 5). Lastly, concerning the correlations between the YW-BCI36 dimensions and the symptoms subscales of the QLQ-BR23, the problems of *body image and sexuality* were negatively correlated with the fact of being *upset by hair loss*, while the problems in the *management of child(ren) and of everyday life* were positively linked with the *therapy side effects* (Table 5).

### Divergent validity

Results showed only one weak correlation between the sharing with close relatives dimension of the YW-BCI36 and the QLQ-C30 and QLQ-BR23 dimensions (*emotional functioning*, r = 0.21), revealing divergent validity in aspects linked with social interactions. Similarly, the *deterioration of the relationships with close relatives* dimension of the YW-BCI36 showed few and weak correlations with the QLQ-BR23 subscales. None of the YW-BCI36 dimension appeared correlated with subscales evaluating specific physical effect sides of the treatments (diarrhea and arm symptoms subscales of the QLQ-BR23) (Table 5).

In summary, as expected we found more correlations (ranging from 0.20 to −0.66) between the dimensions linked with the functioning -whether in the QLQ-C30 or in the QLQ-BR23 module- and the subjective dimensions of the YW-BCI, in particular the *management of child(ren) and of everyday life* and *financial difficulties* dimensions. Less correlations were showed between the symptom dimensions of the QLQ-C30 and QLQ-BR23 and the dimensions of the YW-BCI36 (correlations ranging from −0.22 to 0.58).

## Discussion

The main objective of the present study was to validate, in the French language, a new assessment tool for the impact of non-metastatic breast cancer on the subjective experience of young women (aged under 45 years), which was created from the results of a previous qualitative study [38, 39]. While many studies have already focused on the quality of life of women with breast cancer e.g. [4, 5, 26, 41], taking into account the particular case of young women who have to face specific issues appears crucial for their care.

Following the results of exploratory and confirmatory analyses, the final version of the YW-BCI36 inventory contains 36 items that evaluate 8 types of consequences of the disease and its treatment in terms of impact on daily life for young women living with non-metastatic breast cancer: 1) *feeling of couple cohesion* (5 items), 2) *negative affectivity and apprehension about the future* (6 items), 3) *management of child(ren) and of everyday life* (5 items), 4) *sharing with close relatives* (4 items), 5) *body image and sexuality* (4 items), 6) *financial difficulties* (4 items), 7) *deterioration of relationships with close relatives* (5 items), 8) *career management* (3 items). This 8-factor structure explains 57% of the variance and our study confirms that the YW-BCI36 shows acceptable psychometric properties, with a high level of reliability and validity. There is good internal consistency (above 0.75), and the temporal stability indices are higher than 0.75 for the majority of dimensions. Convergent validity indicates correlations with comparable tools (QLQ-C30 and QLQ-BR23), which is consistent with our hypotheses.

The YW-BCI36 therefore should be considered a complement to existing tools as it addresses not only similar dimensions, but also very specific dimensions, with more precise and targeted item formulations, such as the issues concerning the management of child(ren) in everyday life, positive and negative social relationships with close relatives, and the relationship within the couple. On the other hand, issues concerning symptoms are little depicted in the YW-BCI36 whereas strong correlations are evidenced between its *management of child(ren) and of everyday life* and *career management* dimensions and the symptoms subscales in the QLQ-C30 and QLQ-BR23. Future studies should certainly consider these links in order to refine their determinants and causalities.

The YW-BCI36 was created from the young women’s own subjective experience in order to assess subtly their preoccupations and was validated with a large sample of patients. It makes it possible to identify not only the potential problems of these patients, but also their points of reference/protecting factors, which could help avoid or at least mitigate the impact of the disease and its treatment. This is especially true for dimensions that are related to the sense of cohesion in the couple and to the sharing with close relatives, which appear crucial for the patients’ adjustment when facing cancer e.g. [42–45].

Like the other instruments addressing the quality of life, the YW-BCI36 used in follow-up consultations could also offer patients the opportunity to discuss the issues and challenges that are relevant to them and for which they require psychological and/or medical support. Another advantage of the questionnaire is that it was created and validated with patients at all stages of the care pathway. Among the items that were retained following the patient interviews and psychometric analyses, none is specific to a particular treatment stage, and thus this inventory can be offered to any young woman diagnosed with breast cancer.

Many studies have shown it is relevant to evaluate the quality of life of patients all along the care pathway for modifying their medical care in accordance with their identified needs e.g., [11, 12, 46, 47]. It seemed all the more relevant to create a tool targeting young women with breast cancer to adjust their medical care through an acute evaluation of their subjective experience of the disease and treatments. In addition to its use in the medical practice –allowed by its relatively short form- such a tool could also be used in studies searching to highlight some of the determinants of the quality of life and of its evolution along the care pathway and during the survivorship period in these women.

Nevertheless, future studies should be conducted in a longitudinal design, from the diagnosis announcement to the follow-up period, in order to confirm the stability of the scale and to understand the time evolutions that are closely linked with the situation of each patient during her therapeutic journey. It would also be very interesting to assess the practical and clinical implications of this tool’s use in terms of adapted and personalized support care.

## Conclusions

The YW-BCI36 is a valid measure of the impact of non-metastatic breast cancer on the subjective experience of young women (aged under 45 years). It can be offered to any young woman diagnosed with breast cancer to assess subtly her preoccupations and help the clinicians to improve her care by guiding her toward appropriate support care, throughout her care pathway. The questionnaire is available on request.

## Abbreviations

QLQ-C30: Quality of Life Questionnaire – Cancer 30
QLQ-BR23: Quality of Life Questionnaire – Breast 23
YW-BCI: Young Women with Breast Cancer Inventory
